# A method for managing scientific research project resource conflicts and predicting risks using BP neural networks

**DOI:** 10.1038/s41598-024-59911-w

**Published:** 2024-04-22

**Authors:** Xuying Dong, Wanlin Qiu

**Affiliations:** Institute of Policy Studies, Lingnan University, Tuen Mun, 999077 Hong Kong

**Keywords:** BP neural network, Scientific research project management, Regularization, Resource conflict risk, Deep learning, Computer science, Engineering

## Abstract

This study begins by considering the resource-sharing characteristics of scientific research projects to address the issues of resource misalignment and conflict in scientific research project management. It comprehensively evaluates the tangible and intangible resources required during project execution and establishes a resource conflict risk index system. Subsequently, a resource conflict risk management model for scientific research projects is developed using Back Propagation (BP) neural networks. This model incorporates the Dropout regularization technique to enhance the generalization capacity of the BP neural network. Leveraging the BP neural network’s non-linear fitting capabilities, it captures the intricate relationship between project resource demand and supply. Additionally, the model employs self-learning to continuously adapt to new scenarios based on historical data, enabling more precise resource conflict risk assessments. Finally, the model’s performance is analyzed. The results reveal that risks in scientific research project management primarily fall into six categories: material, equipment, personnel, financial, time, and organizational factors. This study’s model algorithm exhibits the highest accuracy in predicting time-related risks, achieving 97.21%, surpassing convolutional neural network algorithms. Furthermore, the Root Mean Squared Error of the model algorithm remains stable at approximately 0.03, regardless of the number of hidden layer neurons, demonstrating excellent fitting capabilities. The developed BP neural network risk prediction framework in this study, while not directly influencing resource utilization efficiency or mitigating resource conflicts, aims to offer robust data support for research project managers when making decisions on resource allocation. The framework provides valuable insights through sensitivity analysis of organizational risks and other factors, with their relative importance reaching up to 20%. Further research should focus on defining specific strategies for various risk factors to effectively enhance resource utilization efficiency and manage resource conflicts.

## Introduction

In the twenty-first century, driven by rapid technological innovation and a substantial increase in research funding, the number of scientific research projects has experienced exponential growth. These projects, serving as pivotal drivers of scientific and technological advancement, encompass a wide array of domains, including natural sciences, engineering, medicine, and social sciences, among others. This extensive spectrum attracts participation from diverse researchers and institutions^1,2^. However, this burgeoning landscape of scientific research projects brings forth a set of accompanying challenges and predicaments. Foremost among these challenges is the persistent issue of resource scarcity and the diversity of project requirements. This quandary poses a formidable obstacle to the management and execution of scientific research initiatives. It not only impacts the project’s quality and efficiency but can also cast a shadow on an organization’s reputation and the output of its research endeavors^3–5^. For instance, when two university research projects concurrently require the use of a specific instrument with limited availability or in need of maintenance, it may result in both projects being unable to proceed as planned, leading to resource conflicts. Similarly, competition for research funding from the same source can introduce conflicts in resource allocation decisions by the approval authority. These issues are widespread in research projects, and surveys indicate that project delays or budget overruns due to improper resource allocation are common in scientific research. For example, a study on research projects funded by the National Institutes of Health in the United States revealed that approximately 30% of projects faced delays due to improper resource allocation. In Europe, statistics from the European Union’s Framework Programme for Science and Innovation indicate that resource conflicts have impeded about 20% of transnational collaborative research projects from achieving their established research objectives on time. Furthermore, scientific research projects encompass a spectrum of resource requirements essential for their seamless progression, including but not limited to materials, equipment, skilled personnel, adequate funding, and time^6^. The predicament arises when multiple research initiatives necessitate identical or analogous resources simultaneously, creating a challenge for organizations to provide equitable support during peak demand periods. To mitigate the risks associated with resource conflicts, organizations must continually administer their resource allocation and strike a harmonious equilibrium between resource requisites and their availability^7^.

The Back Propagation (BP) neural network, as a prominent deep learning algorithm, boasts exceptional data processing capabilities. Notably, neural networks possess the capacity to swiftly process extensive datasets and extract intricate mapping relationships within data, rendering them versatile tools employed across various domains, including project evaluation, risk assessment, and cost prediction^8,9^. Scientific research project management constitutes a dynamic process. As projects advance and environmental factors evolve, the risk landscape may undergo continuous transformation^10^. The BP neural network’s inherent self-learning ability empowers it to iteratively update its model based on fresh data, enabling seamless adaptation to new circumstances and changes, thereby preserving the model’s real-time relevance^11^. In conclusion, this approach is poised to enhance project management efficiency and quality, mitigate risks, and foster the potential for the successful realization of scientific research projects.

The primary objective of this study is to formulate an evaluation and risk prediction framework for scientific research project management utilizing the BP neural network. This framework aims to address the issues associated with resource discrepancies and conflicts within the realm of scientific research project management. This study addresses the primary inquiry: What types of resource conflict risks exist in scientific research project management? An extensive literature review and empirical data analysis are conducted to answer this question, identifying six main risk categories: materials, equipment, personnel, finance, time, and organizational factors. A comprehensive resource conflict risk index system is constructed based on these categories. To quantitatively assess the importance of different resource conflict risk factors, the Analytic Hierarchy Process (AHP) is employed. This method allowed for the quantification of the influence of each risk factor objectively and accurately by constructing judgment matrices and calculating the weights of each factor. Subsequently, exploration is conducted into the utilization of BP neural networks to construct a resource conflict risk management model for scientific research projects. A BP neural network model is developed incorporating Dropout technology to capture complex correlations between project resource demand and supply. This model self-learns to adapt to new scenarios in historical data, thereby improving prediction accuracy. Research project data is collected from several universities in Xi’an from September 2021 to March 2023 to validate the effectiveness and accuracy of the proposed model. This data is utilized to train and test the model, and its performance is compared with other advanced algorithms such as CNN and BiLSTM. The evaluation is based on two key metrics: accuracy and root mean square error (RMSE), demonstrating excellent fitting ability and prediction accuracy.

The innovation introduced in this study is rooted in the recognition that the proliferation of scientific research initiatives can precipitate resource conflicts and competition, potentially leading to adverse outcomes such as project failure or resource inefficiency. This study harnesses a multi-layer BP neural network as its central computational tool, concomitantly incorporating the establishment of a resource conflict risk index system. This comprehensive model for evaluating and predicting the risks in scientific research project management takes into account both the resource conflict risk index system and the intrinsic characteristics of the BP neural network. This combined approach serves to enhance the efficiency of managing scientific research projects, curtail resource wastage, mitigate the risk of resource conflicts, and ultimately furnish robust support for the enduring success of scientific research endeavors.

## Related work

### Current research landscape in scientific research project management

Scientific research projects hold a pivotal role in advancing scientific and technological frontiers, fostering knowledge generation, and driving innovation. Effective project management in this context ensures the timely delivery, adherence to budgetary constraints, and attainment of predefined quality standards. Numerous scholars have contributed to the body of knowledge concerning scientific research project management. Significant risks in scientific research project management include improper resource allocation, time delays, budget overruns, and collaboration challenges. For instance, concerning time management, Khiat^12^ illustrated that insufficient project planning or external factors often hinder project deadlines. Regarding financial management, Gao^13^ highlighted the lack of transparency in fund allocation and unreasonable budgeting, leading to unnecessary research cost overruns. Previous studies have predominantly concentrated on developing diverse methodologies and tools to identify and assess potential risks in scientific research projects. For instance, quantitative models have been employed by researchers like Jeong et al.^14^ to evaluate project failure probabilities and devise corresponding risk mitigation strategies. Concurrently, Matel et al.^15^ utilized artificial intelligence (AI), including neural networks and machine learning, to conduct comprehensive analyses of project data and predict potential issues throughout project progression.

The preceding studies offer essential groundwork and insights for the scientific research project management discussed in this study. They illuminate key risks encountered in scientific research project management, including inadequate resource allocation, time constraints, budgetary overruns, and collaboration hurdles. These risks are pervasive in scientific research project management, directly impacting project execution efficiency and outcomes. Moreover, these studies furnish empirical data and case studies, elucidating the underlying causes and mechanisms of these risks. For example, the research conducted by Khiat and Gao offers a nuanced understanding of risk factors, enriching the comprehension of the challenges in scientific research project management. Additionally, these studies introduce diverse methods and tools for identifying and evaluating potential risks in scientific research projects. For instance, the works of Jeong et al. and Matel et al. utilize quantitative models and artificial intelligence techniques to comprehensively analyze project data and forecast potential issues in project advancement. These methodologies and tools serve as valuable resources for constructing the research framework and methodologies in this study. Despite the commendable strides made in employing multidisciplinary approaches to address the challenges posed by scientific research project management, the issues related to resource allocation conflicts and quality assurance during project implementation remain fertile ground for future exploration and active investigation.

### Application of BP neural network in project risk and resource management

BP neural networks are renowned for their non-linear fitting and self-learning capabilities, rendering them invaluable for discerning intricate relationships and patterns in project management. Their applications span diverse areas, including resource allocation, risk assessment, schedule forecasting, cost estimation, and more, culminating in heightened efficiency and precision within project management practices. Numerous scholars have ventured into the realm of BP neural network applications within project management. Zhang et al.^16^ introduced a real-time network attack detection method underpinned by deep belief networks and support vector machines. Their findings underscore the method’s potential for bolstering network security risk management, extending novel data security safeguards to scientific research project management. Gong et al.^17^ devised an AI-driven human resources management system. This system autonomously evaluates employee performance and needs, proffering intelligent managerial recommendations. Bai et al.^18^ harnessed BP neural networks to tackle the intricate challenge of selecting service providers for project management portfolios. Leveraging neural networks, they prognosticate the performance of diverse service providers, lending support to project management decision-making. Sivakumar et al.^19^ harnessed BP neural networks to prognosticate the prioritization of production facilities in the bus body manufacturing sector. Their work serves as an illustrative testament to the potential of neural networks in the production and resource allocation facets of scientific project management. Liu et al.^20^ undertook an analysis of the influential factors and early warning signs pertaining to construction workers’ safety conditions. This investigation underscores the profound implications of neural networks in safety management within the context of engineering and construction project management. Li et al.^21^ harnessed optimized BP neural networks to anticipate risks in the financial management arena of listed companies. Their outcomes underscore the utility of neural networks in financial management, providing an exemplar of a risk assessment tool for scientific research project management.

The comprehensive analysis of the aforementioned studies reveals that BP neural networks exhibit substantial capabilities in scrutinizing historical project data, discerning intricate resource demand–supply dynamics, and offering valuable insights for project management decisions and optimizations. These applications underscore the potential of BP neural networks as indispensable tools within the project management domain. Nonetheless, several challenges persist, particularly concerning the real-time adaptability of BP neural networks and their capacity to cater to dynamic project management requisites.

### Research in the field of scientific research project resource management and risk prediction

Within the realm of scientific research project resource management and risk prediction, various studies by notable scholars warrant attention. Jehi et al.^22^ employed statistical models for risk prediction but overlooked the intricate resource conflict relationships within scientific research projects. Efficient project resource management and accurate risk prediction are pivotal for ensuring smooth project execution and attaining desired outcomes. Asamoah et al. elucidated that scientific research projects necessitate both tangible and intangible resources^23^, encompassing materials, equipment, personnel, funding, and time. The judicious allocation and optimal utilization of these resources significantly influence project progress and outcomes. Misallocation of resources can lead to setbacks such as project delays and budget overruns. Meanwhile, Zwikael et al. identified organizational culture, awareness, support, rewards, and incentive programs as key drivers impacting the effective management of scientific research project benefits^24^. These risks can profoundly affect project advancement and outcomes, underscoring the importance of accurate prediction and adept management. Farooq et al. advocated for scientific project management, emphasizing the need for enhanced risk management strategies and management efficacy to foster sustainable enterprise development^25^.

In conclusion, studies on project resource management and risk prediction encompass diverse facets, including resource allocation, risk assessment, and model development. These efforts offer essential theoretical and methodological underpinnings for the effective execution of scientific research endeavors. Given the ongoing expansion and growing complexity of scientific projects, further research on resource management and risk prediction is imperative to navigate increasingly intricate circumstances.

### Summary

A comprehensive review of methods employed in scientific research project management and risk assessment reveals a predominant focus on quantitative analysis, qualitative research, and the integration of AI techniques. In particular, the utilization of BP neural networks, as demonstrated in studies such as Sivakumar et al., Liu et al., and Li et al., underscores their capacity to furnish real-time data analysis and decision-making support for project managers. However, it remains evident that challenges persist in harnessing the full potential of BP neural networks in terms of real-time adaptability and resource allocation within the multifaceted landscape of dynamic project management. Hence, this study accentuates the existing methodological challenges associated with resource conflict resolution, risk management, and overall scientific research project management. Through the optimization and refinement of BP neural network applications in risk assessment, this study strives to furnish organizations with effective decision-making tools. Ultimately, the insights gleaned from this study aim to serve as a valuable reference for scientific research project managers as they navigate the complexities of project risk management.

## Prediction method for scientific research project management risks based on the BP neural network

### Analysis of the construction of a scientific research project management risk system

**Figure 1:**
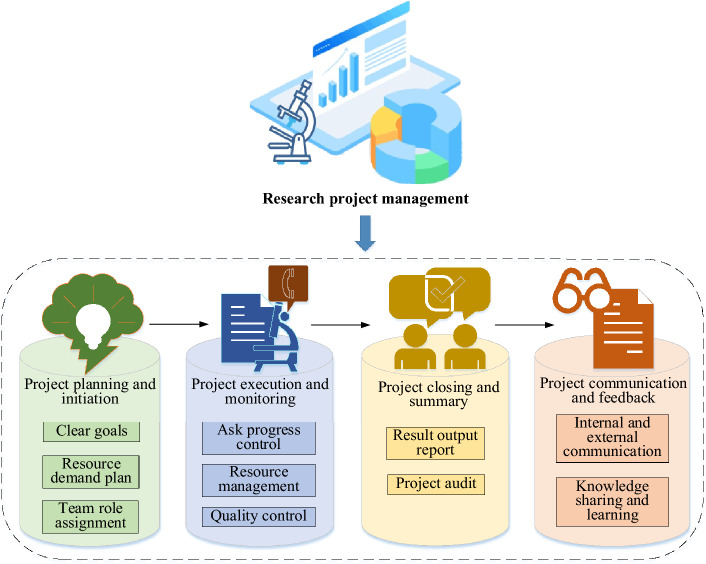
Schematic representation of key scientific research project management tasks.

Scientific research project management constitutes a specialized discipline encompassing the planning, organization, execution, and oversight of scientific research endeavors. Its primary objective is to facilitate the effective attainment of research objectives and anticipated outcomes. The overarching aim of scientific research project management is to optimize resource allocation, schedule planning, and risk mitigation, thereby ensuring the successful culmination of research projects^26,27^. A visual representation of the fundamental task processes integral to scientific research project management is depicted in Fig. 1.

Scientific research project management, as illustrated in Fig. 1, constitutes an essential framework to ensure the efficient and organized execution of scientific research endeavors. It encompasses four core phases: project planning and initiation, project execution and monitoring, project closure and summarization, and project communication and feedback^28^. The meticulous determination of project requisites is of particular significance, encompassing financial resources, personnel, equipment, materials, and more. Failure to ensure the effective utilization and judicious allocation of these resources during project management may introduce the risk of hindrances in the smooth progress and achievement of the research project’s envisioned objectives.

**Figure 2:**
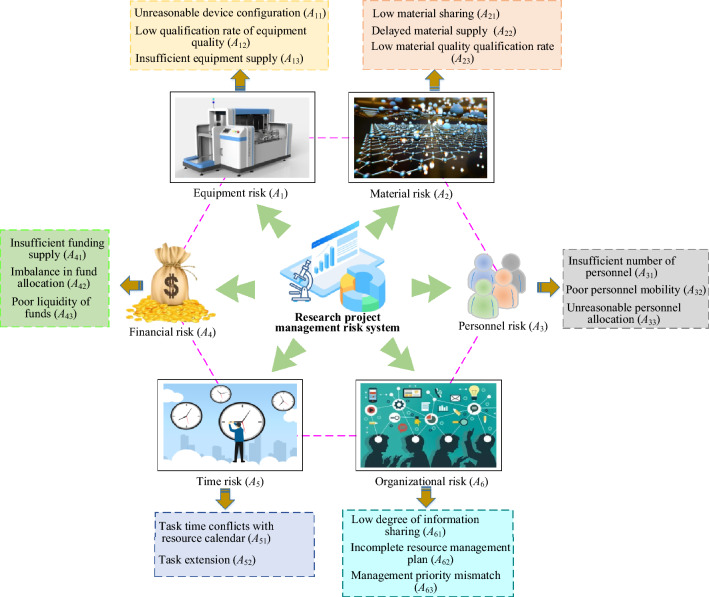
Resource conflict risk indicator system for scientific research project management.

Ongoing scientific research projects necessitate an array of resources, encompassing both tangible assets such as materials, equipment, and funds, and intangible elements like time, personnel expertise, and organizational support^29,30^. These resources are intricately interwoven within scientific research projects and collectively influence project success. However, when confronted with limited total resources, resource conflicts can arise when multiple projects vie for the utilization of the same resources. Consequently, this study has devised a resource conflict risk index system tailored for the management of scientific research projects. This system stratifies risks according to the categories of resources implicated in the project implementation process, as depicted in Fig. 2. In this study, ensuring the representativeness and comprehensiveness of risk assessment for resource conflicts in scientific research project management is pivotal. A multifaceted and systematic approach is adopted to define risk categories. A comprehensive literature review initially identifies common resource conflicts in scientific research project management. Subsequently, through interviews and surveys with industry research project managers, firsthand information on specific challenges and risk factors encountered during project execution is collected. Additionally, referencing international standards and best practices ensures the authority and applicability of risk classification. The outcome of these efforts is illustrated in Fig. 2, showcasing a meticulously designed resource conflict risk index system. It encompasses six major categories: equipment risk, material risk, personnel risk, financial risk, time risk, and organizational risk, further subdivided into 17 specific sub-items. Acknowledging the complexity and diversity of research projects, it is recognized that, despite efforts made, other potential risks may not be included in the current model. A dynamic iterative approach is proposed to address this challenge, integrate additional risk factors, and continuously optimize the model. Specific steps are outlined to enhance the model’s capabilities. Firstly, establishing a monitoring system to regularly collect user feedback and industry updates allows the prompt discovery and incorporation of new risk factors. Simultaneously, closely monitoring the latest research findings in the domestic and international scientific research project management field ensures the continuous integration of new discoveries from academia. Additionally, a dedicated team conducts regular in-depth reviews of the existing risk index system, adding, deleting, or adjusting the weights of risk factors as needed based on actual requirements. This process enables the model to better adapt to the current project management environment and future trends. Secondly, utilizing the newly integrated dataset to cross-validate the model ensures that the newly added risk factors are appropriately assessed and predicted. By comparing the performance of different versions of the model, a more accurate measurement of the effects of optimization is achieved. Finally, research project managers are encouraged to provide real-time feedback, including the model’s performance in actual applications, overlooked risk points, and improvement suggestions, enhancing the model’s usability and reliability. These methods aim to construct a more refined, flexible, and adaptable scientific research project risk assessment model that continuously evolves to meet changing needs. Through continuous optimization and improvement, this model is believed to more effectively assist project managers in making risk-based decisions and promote the success rate of scientific research projects.

As depicted in Fig. 2, this risk system underscores the significance of material quality and timely supply in project execution. The establishment of this resource conflict risk indicator system forms a fundamental basis for subsequent model development and risk forecasting, empowering project managers to gain comprehensive insights into and effectively manage resource conflict risks.

### Weight analysis process using APH for the risk indicator system

**Figure 3:**
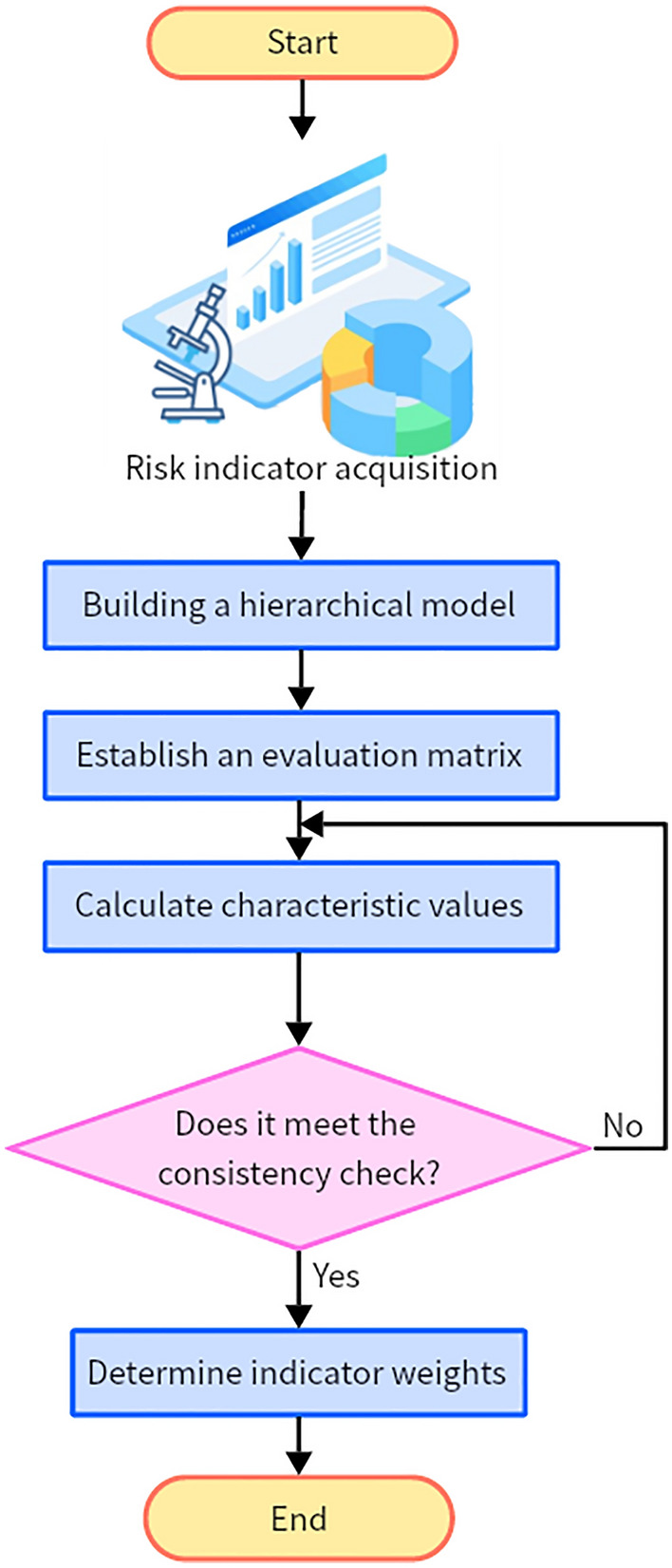
Weight analysis process of applying the hierarchical analysis method to the risk indicator system.

The AHP is primarily employed for the comprehensive analysis of multifaceted problem systems, involving the segmentation of interrelated factors into hierarchical levels. It subsequently facilitates objective assessments at each tier. This method typically deconstructs problems into a tripartite structure comprising the following levels: the objective layer (highest), the criteria layer (intermediate), and the indicator layer (fundamental)^31,32^. In this context, the objective layer pertains to the project’s resource conflict risk, which represents the core challenge addressed by this structural model. The criteria layer provides an initial decomposition of the objective layer and establishes the foundational logical framework for third-level indicators. The indicator layer encompasses risk factors, specifically, the potential triggers for resource conflict risks. The weight analysis process, employing the AHP for the risk indicator system, is delineated in Fig. 3.

In Fig. 3, the application of the AHP to the weight analysis of the scientific research project management risk indicator system follows a general procedure: sequentially defining individual problems, creating a hierarchical structural model, constructing pairwise comparison matrices, performing hierarchical ranking calculations and consistency tests, and finally, selecting evaluation criteria systematically for assessment.

The initial step involves breaking down the intricate problem into distinct components, creating a hierarchical structure model comprising the target layer, criterion layer, and indicator layer.

In this phase, the assessment of relative importance between elements leads to the formation of a pairwise comparison judgment matrix, denoted as matrix *A*, as depicted in Eq. (1).

1 $$A = \left( {a_{ij} } \right)_{n \times n}$$

In Eq. (1), $a_{ij} > 0$, $a_{ji} = 1/a_{ij}$, and $a_{ii} = 1$.

The AHP calculations are performed following the classic methodology proposed by Rehman^33^. The process begins by computing the product *M*_*i*_ of the elements within each row, as illustrated in Eq. (2).

2 $$M_{i} = \prod\limits_{j = 1}^{n} {a_{ij} }$$

The next step involves calculating the *n*-th root of *M*_*i*_, as described in Eq. (3).

3 $$W_{i} = \sqrt[n]{{M_{i} }}$$

Next, the process involves normalizing $W = \left[ {W_{1} ,W_{2} , \cdots ,W_{n} } \right]^{T}$, as shown in Eq. (4).

4 $$w_{i} = \frac{{W_{i} }}{{\sum\nolimits_{i = 1}^{n} {W_{i} } }}$$

Finally, the maximum eigenvalue $\lambda_{\max }$ is calculated via Eq. (5).

5 $$w_{i} = \frac{{W_{i} }}{{\sum\nolimits_{i = 1}^{n} {W_{i} } }}$$

The calculation of weights and the consistency test of the judgment matrix involve the use of the eigenvalue method to calculate the weight vector of the judgment matrix. This is demonstrated in Eq. (6).

6 $$AQ = \lambda_{\max } Q$$

In Eq. (6), $\lambda_{\max }$ denotes the maximum characteristic root of *A*,* Q* signifies the eigenvector, and the weight vector is obtained by normalizing *Q.*

Continuing with the consistency testing, the weight vector must undergo evaluation for consistency. To initiate this evaluation, calculate the Consistency Index (*C.I.*) using Eq. (7).

7 $$C.I. = \frac{{\lambda_{\max } - n}}{n - 1}$$

Next, it is imperative to determine the corresponding average Random Consistency Index (*R.I.*). Subsequently, the Consistency Ratio (*C.R.*) is computed using the formula presented in Eq. (8).

8 $$C.R. = \frac{C.I.}{{R.I.}}$$

If the calculated *C.R.* is less than 0.1, it indicates that the judgment matrix meets the prescribed consistency criteria, and the assigned weight values for each indicator are considered valid. However, if the calculated *C.R.* equals or exceeds 0.1, this signals the need for adjustments to the judgment matrix. To address this, the matrix is re-evaluated, and consistency checks are repeatedly performed until the matrix achieves the required level of consistency.

### Analyzing the resource conflict risk management model for scientific research projects based on the BP neural network

**Figure 4:**
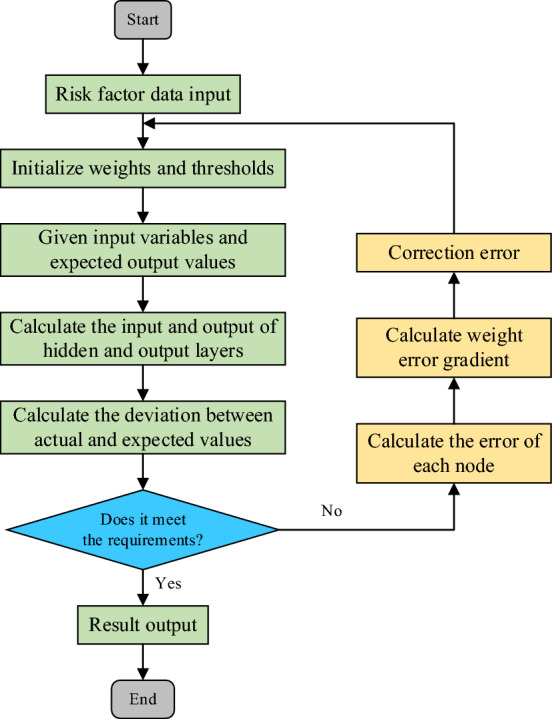
Data transmission flow chart of the BP neural network.

This section focuses on predicting and evaluating the potential occurrence of various risk factors within scientific research projects. The objective is to facilitate the selection of appropriate response strategies aimed at minimizing losses stemming from risks associated with scientific research endeavors. Resource management within scientific research projects is a complex undertaking, with resource conflict risks influenced by a multitude of factors. Furthermore, as projects evolve, the risk landscape undergoes dynamic changes. In contrast to conventional statistical models, BP neural networks offer distinctive advantages. They employ a combination of forward signal propagation and reverse error-adjustment learning techniques, showcasing exceptional self-learning capabilities, distributed knowledge storage, and associative memory functions^34^. The BP neural network model, rooted in the backpropagation algorithm, evolved from the necessity to simulate biological neural systems and meet the demands of machine learning. Originating in the 1980s, it became a prominent deep learning model, continually iterating and adjusting connection weights to minimize the error between output and target. This learning mechanism allows the BP neural network to adapt to complex non-linear relationships, showcasing robust approximation and generalization capabilities. Over time, enhanced computer hardware and algorithm optimization led to widespread application and development of the BP neural network model. Algorithmically, various improvements, including the momentum method, adaptive learning rate, and regularization, were introduced to boost training speed and generalization ability, addressing challenges such as susceptibility to local minima in traditional BP algorithms. The advent of deep learning saw the integration of the BP neural network into deeper structures like ResNet and CNN, enabling it to handle more intricate tasks and data. The model’s applicability expanded across diverse domains, including image and speech recognition, natural language processing, financial forecasting, and medical diagnosis, yielding breakthrough results. Moreover, technological advancements like big data and cloud computing have enhanced the training and application efficiency of the BP neural network model, presenting new avenues for development. In conclusion, the evolution of the BP neural network model stems from algorithmic refinements, structural enhancements, and broadened applications, providing potent tools for addressing diverse practical challenges. The data transmission process of the BP neural network is illustrated in Fig. 4.

Figure 4 illustrates the data transmission process in the BP neural network, highlighting forward propagation, which entails processing and transmitting received data information. This unidirectional propagation begins at the input layer, traverses through the hidden layers, and culminates in the output layer to yield the network’s overall output. Let the received input data be denoted as *X* = (*x*_1_, *x*_2_…, *x*_*n*_), with ‘*n*’ signifying the number of neurons in the input layer. The connections between the input layer and the hidden layer initially possess randomized weight values. This citation is derived from Liu et al.’s recommendation^35^ to prevent premature convergence to local minima during the training process. Representing the weight of the connection between the *i*-th neuron in the input layer and the *j*-th neuron in the hidden layer as *W*_*ij*_. The notation follows Narkhede et al.’s study^36^, which offers a comprehensive explanation of neural network fundamentals and operational principles. The information received by the hidden layer is expressed in Eq. (9).

9 $$a_{j} = \sum\limits_{i} {x_{i} w_{ij} }$$

In Eq. (9), *i* represents the number assigned to neurons in the input layer, ‘*j*’ pertains to the number of neurons in the hidden layer, and *A* = (*a*_1_, *a*_2_…, *a*_*m*_) symbolizes the input variables received by the hidden layer. Upon receiving these variables, the hidden layer neuron transforms them into the output value of the hidden layer using the activation function. The methodology in this section draws from the research by Narengbam et al.^37^ on activation functions in deep learning models. Specifically, the treatment of the output layer mirrors that of the hidden layers, and the computation of output layer neurons adheres to the methodology outlined in the cited literature.

10 $$y_{j} = f\left( {a_{j} } \right) = \frac{1}{{1 + e^{{ - a_{j} }} }}$$

In Eq. (10), *Y* = (*y*_1_, *y*_2_…, *y*_*m*_) represents the output variables of the hidden layer. The computation method for the input and output values of the output layer parallels that of the hidden layer. The weight denoted as *v*_*jk*_ signifies the connection between the *j*-th neuron in the hidden layer and the *k*-th neuron in the output layer. The information received by the output layer is described in Eq. (11).

11 $$b_{k} = \sum\limits_{j} {y_{j} v_{jk} }$$

The output value of the output layer neurons, once activated by the activation function, is expressed in Eq. (12).

12 $$o_{k} = f\left( {b_{k} } \right) = \frac{1}{{1 + e^{{ - b_{k} }} }}$$

At this juncture, the output value *O* denoted as $O = \left( {o_{1} ,o_{2} , \cdots ,o_{z} } \right)$ is obtained, signifying the conclusion of the forward propagation process.

In the backpropagation process, the loss function *J* quantifies the error between the neural network’s output value and the true value (referring to the definition and application of the loss function in neural network optimization as articulated by Özden et al.^38^), as illustrated in Eq. (13).

13 $$J\left( {W,b;x,y} \right) = \frac{1}{2}\left\| {h_{W,b} \left( x \right) - y} \right\|^{2}$$

During the neural network’s training process, the weight, denoted as *W*, and the bias vector, denoted as *b*, play essential roles. The gradient descent method is employed to optimize the neural network (derived from Kumar et al.’s^39^ analysis of the effectiveness of optimization algorithms in deep learning training). Each iteration within the gradient descent method updates the parameters *W* and *b* as per Eqs. (14) and (15).

14 $$W_{ij}^{\left( l \right)} = W_{ij}^{\left( l \right)} - \alpha \frac{\partial }{{\partial W_{ij}^{\left( l \right)} }}J\left( {W,b} \right)$$

15 $$b_{i}^{\left( l \right)} = b_{i}^{\left( l \right)} - \alpha \frac{\partial }{{\partial b_{i}^{\left( l \right)} }}J\left( {W,b} \right)$$

where *α* represents the learning rate. The crucial step involves computing derivatives using backpropagation, employing the BP algorithm to calculate $\frac{\partial }{{\partial W_{ij}^{\left( l \right)} }}J\left( {W,b;x,y} \right)$ and $\frac{\partial }{{\partial b_{i}^{\left( l \right)} }}J\left( {W,b;x,y} \right)$. These two components represent the derivatives of the cost function *J*(*W*, *b*; *x*, *y*) for a single sample (*x*, *y*). Once this derivative is computed, deriving the derivatives of the overall cost function *J*(*W*, *b*; *x*, *y*) becomes relatively straightforward. The calculated results are presented in Eqs. (16) and (17).

16 $$\frac{\partial }{{\partial W_{ij}^{\left( l \right)} }}J\left( {W,b} \right) = \left[ {\frac{1}{m}\sum\limits_{i = 1}^{m} {\frac{\partial }{{\partial W_{ij}^{\left( l \right)} }}J\left( {W,b;x^{\left( i \right)} ,y^{\left( i \right)} } \right)} } \right] + \lambda W_{ij}^{\left( l \right)}$$

17 $$\frac{\partial }{{\partial b_{i}^{\left( l \right)} }}J\left( {W,b} \right) = \frac{1}{m}\sum\limits_{i = 1}^{m} {\frac{\partial }{{\partial W_{ij}^{\left( l \right)} }}J\left( {W,b;x^{\left( i \right)} ,y^{\left( i \right)} } \right)}$$

This study aims to develop a resource conflict risk management model tailored to predict and assess the resource conflict risks inherent in scientific research projects during execution. Resource conflicts arise from competition for limited resources like equipment, funding, and personnel among multiple projects. If unaddressed, these conflicts can significantly impede project progress and outcomes. The model’s specific objectives are to analyze project-related information (e.g., project scale, duration, funding, personnel allocation) to predict potential conflict points in resource allocation, enabling project managers to proactively mitigate or avoid conflicts and optimize resource utilization effectively. To achieve these objectives, we employ a BP neural network approach for model construction, chosen for its superior non-linear mapping capability and self-learning characteristics, enabling it to learn from extensive historical project data and identify complex resource conflict risk patterns. The model construction entails key steps: Data preprocessing involves cleaning and normalizing collected project data to meet model input requirements. Feature selection entails choosing highly correlated feature variables associated with resource conflict risks as model inputs based on expert knowledge and data analysis results. Model training and validation involve training the BP neural network with labeled historical project data and evaluating and optimizing model performance through techniques like cross-validation. Through these methods, the developed model accurately predicts resource conflict risks in scientific research project management, providing decision support for project managers to enhance resource utilization efficiency and foster successful project completion.

**Figure 5:**
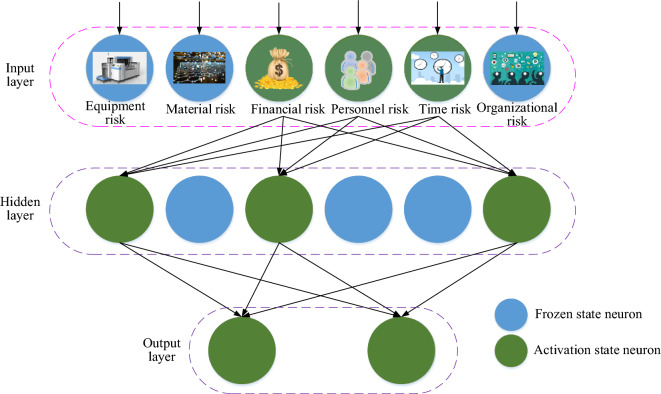
Network topology based on the BP neural network applied to the resource conflict risk management model for scientific research projects.

While the BP neural network possesses robust learning and non-linear fitting capabilities, inadequate training data can lead to suboptimal fitting. In some cases, the network may only excel at learning from a limited dataset, generating a mapping function (typically represented as a weight vector) that closely matches the training dataset. Consequently, it may struggle to generalize well to new data, exhibiting insufficient generalization abilities. This scenario is known as overfitting. To mitigate overfitting, this study introduces the Dropout regularization method^40^ when applying the BP neural network to scientific research project risk management. The Dropout method involves freezing nodes within the input and hidden layers. It is particularly useful when specific neuron correlations in the input layer hinder continuous error convergence during training. The node freezing rate should strike a balance—not too low, as it would have an insignificant impact on the neural network, and not too high, which could lead to underfitting. Therefore, this study sets the node freezing rate for the Dropout regularization method at 50%. By incorporating the Dropout method into the BP neural network, the network topology used for managing resource conflict risks in scientific research projects, based on the BP neural network, is depicted in Fig. 5.

As depicted in Fig. 5, this model incorporates a novel approach. During each training iteration, a randomly selected set of neurons, encompassing those associated with equipment, materials, and organizational risk factors, is temporarily frozen. These frozen neurons do not participate in either the forward propagation calculations or the subsequent backpropagation error adjustments within the current training cycle. The weights connecting these neurons to others retain their previous states or revert to their initial values from the last training update. As the next training iteration commences, the neurons previously frozen are unfrozen, and a new batch of neurons is randomly chosen for freezing. This iterative process effectively bolsters the BP neural network’s ability to generalize from limited data, particularly when addressing resource conflict risk management in research projects.

**Figure 6:**
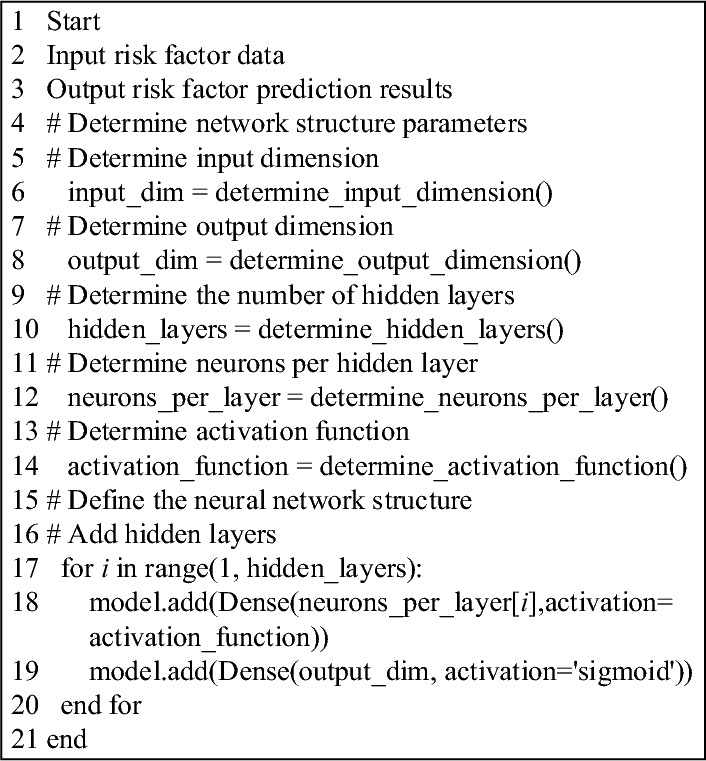
Flowchart presenting the pseudocode algorithm for optimizing the BP neural network.

The integration of the Dropout method into the BP neural network introduces further opportunities for optimization. Adjustments to the network’s depth, the number of neurons, and the choice of activation functions within the risk prediction model can be made. The specific optimization procedure for the BP neural network is outlined in Fig. 6.

### Experimental evaluation

To assess the performance of the resource conflict risk management model developed in this study, a BP neural network was constructed utilizing the ‘newff’ function within MATLAB. Python was employed for data preprocessing and algorithm implementation. The training of the BP neural network involved configuring parameters for *net.trainFcn* and *net. trainParam* following network initialization. Training iterations continued until the error met the predefined performance criterion. The dataset utilized in this study consisted of research project information spanning all universities in Xi’an, China, from September 2021 to March 2023. In comprehensively evaluating the performance of the resource conflict risk management model developed in this study, the scope and objectives of data collection are first determined, focusing primarily on scientific research projects at major universities in the Xi’an area. Data sources included publicly available project records, official website information, and pertinent research project databases. The utilization of web scraping techniques facilitates automated data collection, encompassing details such as project names, principal investigators, start and completion dates, funding particulars, research areas, and participating personnel. Rigorous anonymization and encryption measures are implemented to uphold information security. Subsequently, to enhance understanding of the data characteristics, exploratory data analysis is conducted on the cleaned dataset. This involves calculating descriptive statistics, conducting distribution tests, and performing correlation analysis. Such steps aid in identifying the most influential feature variables for the predictive model. Given that raw data often contain missing values, outliers, or inconsistencies, comprehensive data cleaning is executed, which includes imputation of missing values, removal of outlier data, and standardization of data formats. To safeguard individual privacy, sensitive information such as project leader names undergoes anonymization and encryption. Concerning the application of the AHP in this study, this method is employed to ascertain the relative weights of various risk factors (including materials, equipment, funding, time, personnel skills, and organizational support). The operational process involves establishing a pairwise comparison judgment matrix based on expert assessments and historical data analysis. Each element in the matrix reflects the importance of one risk factor relative to another. The weights of each risk factor are determined by calculating the maximum eigenvalue of the judgment matrix and its corresponding eigenvector. Consistency indices and random consistency ratios are used to verify the consistency of the judgment matrix, deeming the derived weights acceptable only when the random consistency ratio is below 0.1. Using these meticulously assigned weighted risk factors throughout the model evaluation process, resource conflict risk prediction is conducted via the BP neural network using data collected from actual scientific research projects.

Subsequently, rigorous data anonymization procedures were applied, including de-identification, data anonymization, and encryption of sensitive information. The data preprocessing workflow encompassed comprehensive data cleaning to rectify missing or outlier data points. Ultimately, data from 8,175 research projects were amassed and segregated into training and testing subsets, with an 80% to 20% partition ratio.

To assess the performance of the model developed in this study, an initial step involved employing the AHP to evaluate the weights assigned to each factor, including materials, equipment, funds, time, personnel skills, and organization. Subsequently, the algorithm presented in this study was combined with the Convolutional Neural Network (CNN)^41^, Bidirectional Long Short-Term Memory (BiLSTM)^42^, and comparative experiments were conducted in alignment with recent studies conducted by Liu et al. and Li et al. The evaluation primarily relied on accuracy and RMSE as key metrics, precisely measuring model prediction accuracy. Additionally, the Garson sensitivity analysis method was employed to assess the sensitivity of risk factors across various algorithms.

## Results and discussions

### Analysis of weights and sensitivity results of different factors

**Figure 7:**
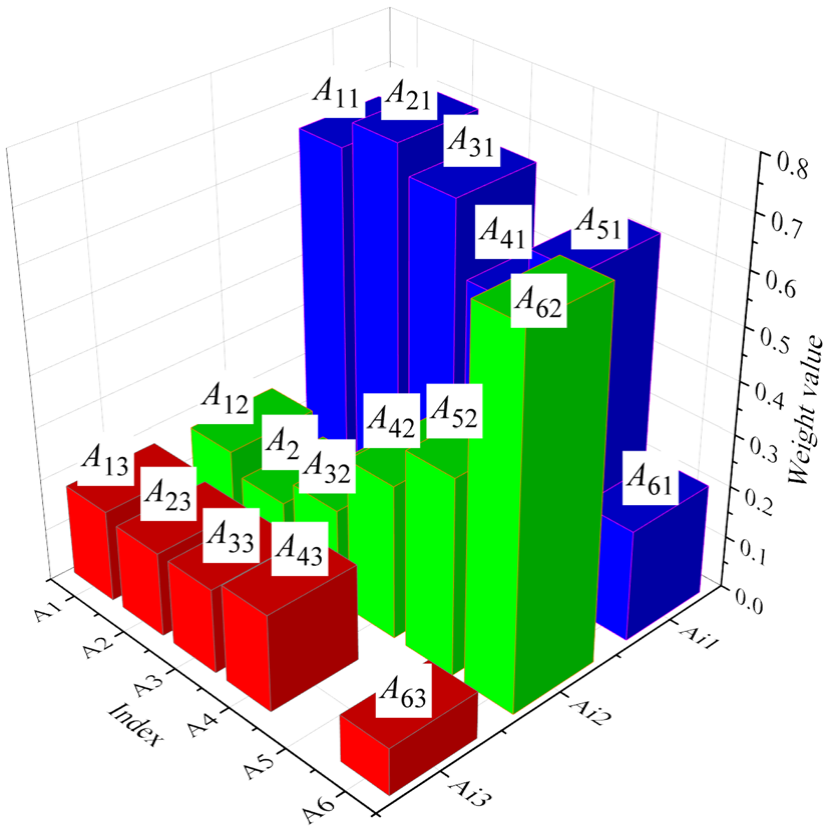
Weight results of different factors.

**Figure 8:**
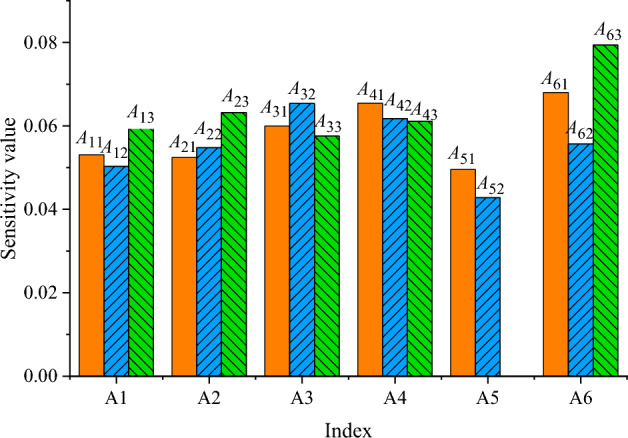
Sensitivity results of different factors.

The analysis of weights and sensitivities for various factors is depicted in Figs. 7 and 8.

Figure 7 highlights the various risk factors present in scientific research project management, including materials, equipment, funds, time, personnel skills, and organization. A more in-depth examination of the weight of sub-indicators within each factor reveals that *A*_*21*_ holds the highest weight value, at 0.705, while *A*_*63*_ carries the smallest weight value. Consequently, the application of the AHP in this study enables a clear representation of the significance of each influencing factor. This, in turn, facilitates a more targeted and informed decision-making process, allowing for decisions that align better with the actual circumstances and desired outcomes.

Figure 8 reveals notable variations in the sensitivity of each risk factor to the model’s output variables. Organizational risk emerges as the most influential factor on the comprehensive risk value, accounting for a relative importance of 20.31%. Following closely are financial risk at 18.84%, personnel risk at 18.30%, material risk at 17.04%, equipment risk at 16.29%, and time risk at 9.24%. A more detailed scrutiny of the sensitivity of individual sub-indicators within each factor uncovers that A52 exhibits the lowest sensitivity, standing at 4.28%, while A63 records the highest sensitivity, reaching 7.84%.

### Model performance comparison results under different algorithms

**Figure 9:**
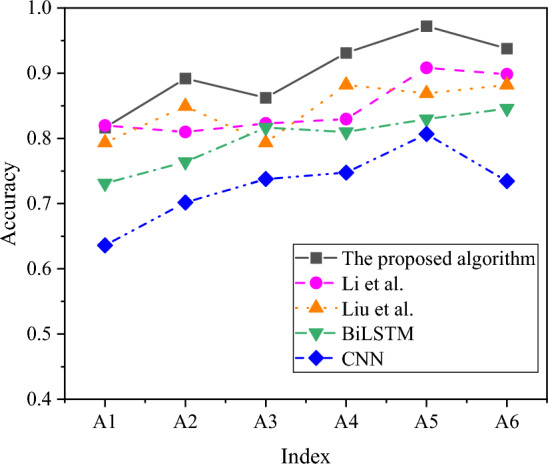
Visual representation of accuracy results achieved by different algorithms across various factors.

**Figure 10:**
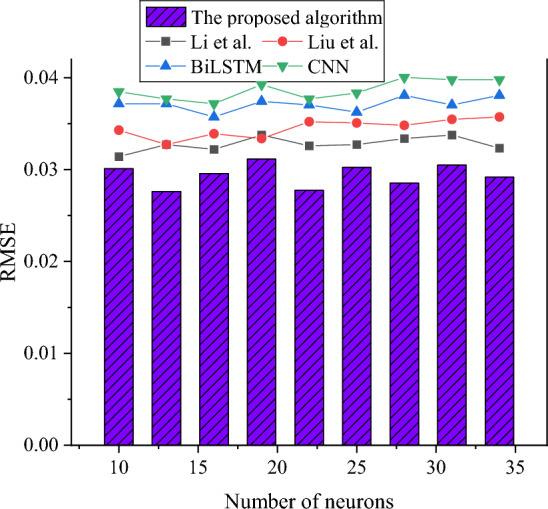
RMSE comparison results of each algorithm under different numbers of neurons.

In-depth analysis encompassed evaluating the accuracy and RMSE outcomes of distinct algorithms across diverse indicators, as depicted in Figs. 9 and 10.

Figure 9 illustrates that the accuracy of various algorithms remains relatively stable across different index factors. Notably, the risk prediction accuracy achieved by the algorithm proposed in this study outperforms other model algorithms across various factors. The highest risk prediction accuracy is observed in the time factor, reaching an impressive 97.21%, while the equipment factor yields the lowest prediction accuracy, hovering around 80%. Upon further comparison of risk prediction accuracy across algorithms, it becomes evident that the model algorithms proposed in this study outperform Li et al.’s model algorithm and Liu et al.’s model algorithm. Additionally, the proposed model algorithm surpasses BiLSTM and CNN. Consequently, this study’s model algorithm effectively identifies risk factors in the management of scientific research projects.

Figure 10 presents the RMSE results of each algorithm, and it is evident that increasing the number of hidden layer neurons does not significantly alter the RMSE values. Specifically, the RMSE of the model algorithm introduced in this study consistently remains around 0.03. In contrast, other model algorithms yield RMSE values exceeding 0.031, indicating higher errors compared to the model proposed in this study. When arranging the RMSE results in ascending order, it becomes apparent that the order is as follows: the model algorithm introduced in this study has the lowest RMSE, followed by Li et al.’s proposed model algorithm, Liu et al.’s proposed model algorithm, BiLSTM, and CNN. Therefore, the research model demonstrates effective risk prediction in scientific research project management, characterized by lower identification errors and superior fitting capabilities.

## Conclusion

This study established a resource conflict risk index system for scientific research project management and introduced a BP neural network as a risk prediction model. Leveraging its non-linear fitting and self-learning capabilities, the model effectively captured intricate resource demand and supply dynamics, enabling a more precise assessment of resource conflict risks. The performance evaluation revealed the model’s strength in predicting time-related risks, achieving an accuracy rate of 97.21% with an RMSE consistently around 0.03, indicating strong fitting capabilities. The developed BP neural network model in this study effectively predicts resource conflict risks in scientific research project management, serving as a valuable decision support tool for risk assessment. However, certain limitations are acknowledged in this research. Firstly, the dataset is derived from universities in a specific region (Xi’an), and although sizable, it may not comprehensively represent all types of scientific research projects. Future endeavors could involve incorporating more diverse and extensive data sources to enhance the model’s universality and robustness. Secondly, despite the notable advantages of BP neural networks in addressing non-linear problems, the selection of appropriate network structures and parameter settings remains a challenge. Subsequent work could focus on further enhancing the network’s performance through the exploration of additional optimization algorithms. In terms of future research directions, the following points are proposed: Firstly, considering the integration of various machine learning and deep learning technologies to obtain more comprehensive risk prediction results. Secondly, exploring the application of the model in scientific research projects of different scales and types to validate and broaden its applicability. Lastly, investigating the integration of the model into a real-time project management system can provide project managers with dynamic risk monitoring and warning services.

### Supplementary Information

Supplementary Information.

## Data Availability

All data generated or analysed during this study are included in this published article [and its supplementary information files].

## References

[1] Ren S, Li L, Han Y (2022). The emerging driving force of inclusive green growth: Does digital economy agglomeration work?. Bus. Strateg. Environ..

[2] Wang W, Hu Y, Lu Y (2023). Driving forces of China’s provincial bilateral carbon emissions and the redefinition of corresponding responsibilities. Sci. Total Environ..

[3] Do ST, Nguyen VT, Likhitruangsilp V (2023). RSIAM risk profile for managing risk factors of international construction joint ventures. Int. J. Constr. Manag..

[4] Nguyen HD, Do QNH, Macchion L (2023). Influence of practitioners’ characteristics on risk assessment in Green Building projects in emerging economies: A case of Vietnam. Eng. Constr. Archit. Manag..

[5] Shayan S, Pyung Kim K, Tam VWY (2022). Critical success factor analysis for effective risk management at the execution stage of a construction project. Int. J. Constr. Manag..

[6] Alam I, Sarwar N, Noreen I (2022). Statistical analysis of software development models by six-pointed star framework. PLoS ONE.

[7] Pham HT, Pham T, Truong Quang H (2023). Supply chain risk management research in construction: A systematic review. Int. J. Constr. Manag..

[8] Zhao Y, Chen W, Arashpour M (2022). Predicting delays in prefabricated projects: SD-BP neural network to define effects of risk disruption. Eng. Constr. Archit. Manag..

[9] Zhang X, Li W, Zhang X (2023). Application of grey feed forward back propagation-neural network model based on wavelet denoising to predict the residual settlement of goafs. PLoS ONE.

[10] El Khatib M, Al Mulla A, Al KW (2022). The role of blockchain in E-governance and decision-making in project and program management. Adv. Internet Things.

[11] Ujong JA, Mbadike EM, Alaneme GU (2022). Prediction of cost and duration of building construction using artificial neural network. Asian J. Civil Eng..

[12] Khiat H (2022). Using automated time management enablers to improve self-regulated learning. Act. Learn. High. Educ..

[13] Gao J (2022). Analysis of enterprise financial accounting information management from the perspective of big data. Int. J. Sci. Res..

[14] Jeong J, Jeong J (2022). Quantitative risk evaluation of fatal incidents in construction based on frequency and probability analysis. J. Manag. Eng..

[15] Matel E, Vahdatikhaki F, Hosseinyalamdary S (2022). An artificial neural network approach for cost estimation of engineering services. Int. J. Constr. Manag..

[16] Zhang H, Li Y, Lv Z (2020). A real-time and ubiquitous network attack detection based on deep belief network and support vector machine. IEEE/CAA J. Autom. Sin..

[17] Gong Y, Zhao M, Wang Q (2022). Design and interactive performance of human resource management system based on artificial intelligence. PLoS ONE.

[18] Bai L, Zheng K, Wang Z (2022). Service provider portfolio selection for project management using a BP neural network. Ann. Oper. Res..

[19] Sivakumar A, Singh NB, Arulkirubakaran D (2023). Prediction of production facility priorities using Back Propagation Neural Network for bus body building industries: A post pandemic research article. Qual. Quant..

[20] Liu N, Xie D, Wang C (2023). Influencing factors and prewarning of unsafe status of construction workers based on BP neural network. Appl. Sci..

[21] Li X, Wang J, Yang C (2023). Risk prediction in financial management of listed companies based on optimized BP neural network under digital economy. Neural Comput. Appl..

[22] Jehi L, Ji X, Milinovich A (2020). Individualizing risk prediction for positive coronavirus disease 2019 testing: Results from 11,672 patients. Chest.

[23] Asamoah RO, Baiden BK, Nani G (2022). Identifying intangible resources to enhance profitability strategies of Small-Medium Scale Construction Firms (SMSCFs) in developing countries. Int. J. Construct. Manag..

[24] Zwikael O, Huemann M (2023). Project benefits management: Making an impact on organizations and society through projects and programs. Int. J. Project Manag..

[25] Farooq R (2024). A review of knowledge management research in the past three decades: A bibliometric analysis. VINE J. Inf. Knowl. Manag. Syst..

[26] Bergevin MD, Ng V, Menzies P (2023). Cache a Killer: Cache Valley virus seropositivity and associated farm management risk factors in sheep in Ontario, Canada. PLoS ONE.

[27] Huang G, Lee SM, Clinciu DL (2023). Competitive advantages of organizational project management maturity: A quantitative descriptive study in Australia. PLoS ONE.

[28] Yesica R, Jerahmeel G, Ichsan M (2023). Project management office manager’s competences: Systematic literature review. Int. J. Project Organ. Manag..

[29] Yu C, Hsiao YC (2022). IT project management resource: Identifying your project’s common goals. Int. J. Inf. Technol. Project Manag..

[30] Qu S, Chen H, Shen Z (2023). The performance evaluation of management mode of small water resources projects. PLoS ONE.

[31] Wu Z, Xue W, Xu H (2022). Urban flood risk assessment in Zhengzhou, China, based on a D-number-improved analytic hierarchy process and a self-organizing map algorithm. Remote Sens..

[32] Lin CL, Fan CL, Chen BK (2022). Hybrid analytic hierarchy process-artificial neural network model for predicting the major risks and quality of Taiwanese construction projects. Appl. Sci..

[33] Rehman A, Song J, Haq F (2022). Multi-hazard susceptibility assessment using the analytical hierarchy process and frequency ratio techniques in the Northwest Himalayas, Pakistan. Remote Sens..

[34] Liu J, Zhan C, Wang H (2023). Developing a hybrid algorithm based on an equilibrium optimizer and an improved backpropagation neural network for fault warning. Processes.

[35] Narkhede MV, Bartakke PP, Sutaone MS (2022). A review on weight initialization strategies for neural networks. Artif. Intell. Rev..

[36] Narengbam L, Dey S (2023). Harris hawk optimization trained artificial neural network for anomaly based intrusion detection system. Concurr. Comput. Pract. Exp..

[37] Özden A, İşeri İ (2023). COOT optimization algorithm on training artificial neural networks. Knowl. Inf. Syst..

[38] Kumar G, Singh UP, Jain S (2022). Swarm intelligence based hybrid neural network approach for stock price forecasting. Comput. Econ..

[39] Zhao Y (2023). Application of BP neural network algorithm in visualization system of sports training management. Soft Comput..

[40] Nketiah EA, Chenlong L, Yingchuan J (2023). Recurrent neural network modeling of multivariate time series and its application in temperature forecasting. PLoS ONE.

[41] Kumar TA, Rajmohan R, Adeola Ajagbe S (2023). A novel CNN gap layer for growth prediction of palm tree plantlings. PLoS ONE.

[42] Liu J, Cao JX, Ding WN (2022). Research on reservoir porosity prediction method based on bidirectional longshort-term memory neural network. Prog. Geophys..

