# Supplementary material for: A method for managing scientific research project resource conflicts and predicting risks using BP neural networks
Source: Sci Rep. 2024 Apr 22;14:9238. doi: 10.1038/s41598-024-59911-w (PMC11035660; doi:10.1038/s41598-024-59911-w)
Supplement: Supplementary file 1 — Supplementary Information. [file 41598_2024_59911_MOESM1_ESM.zip › Data packet/Code description and configuration.docx]

**Code Description:**

**Importing Necessary Libraries and Modules:** Initially, the code imports the TensorFlow library, a commonly used framework for developing and training deep learning models.

**Determining Network Structure Parameters:** This section involves defining structural parameters for the neural network. Functions such as ‘determine_input_dimension()’, ‘determine_output_dimension()’, ‘determine_hidden_layers()’, ‘determine_neurons_per_layer()’, and ‘determine_activation_function()’ are called to set parameters like input dimension, output dimension, the number of hidden layers, neurons per layer, and activation function. These parameters are typically tailored to the specific problem.

**Defining Neural Network Structure:** The code creates a sequential model using ‘Sequential()’ nd adds various layers to the neural network with ‘model.add()’. It begins with an input layer, proceeds to add the specified number of hidden layers within a loop, and concludes with an output layer. Each hidden layer contains the defined number of neurons and employs the chosen activation function. A Dropout layer is inserted after the input layer to combat overfitting.

**Compiling the Model:** This step involves compiling the neural network model through ‘model.compile()’. The compilation process includes specifying the optimizer (in this case, the Adam optimizer), the loss function (binary cross-entropy loss for binary classification problems), and the performance metric to be monitored (accuracy in this instance).

**Preparing Training Data and Labels:** Using the ‘prepare_training_data()’ function, training data (X_train) and corresponding labels (y_train) are prepared.

**Training the Model:** The model is trained using ‘model.fit()’. Parameters such as the number of training epochs, batch size for each iteration, and the validation set split ratio are specified. The model repeatedly iterates over the training data, continuously adjusting weights to minimize the loss function.

**Risk Prediction:** Finally, by employing prepare_testing_data(), the code prepares the test data (X_test), and the trained model is utilized for prediction to yield the predicted results (y_pred).

**Configuration:**

Configuration functions are employed to specify essential parameters of the neural network model and data preparation process. This study encompasses network structural parameters, including input dimension, output dimension, the number of hidden layers, the number of neurons per layer, and the activation function.

determine_input_dimension():input_dim = len(selected_features);

determine_output_dimension():output_dim = 1;

determine_hidden_layers():hidden_layers = 12;

determine_neurons_per_layer():neurons_per_layer = [64, 128, 64];

determine_activation_function():activation_function = ' sigmoid';

**How to Use the Algorithm:**

The algorithm model can be employed by following these steps:

1. Configure the network structure parameters in the code.
2. Define and implement data preparation functions to prepare training and testing data.
3. Compile the model, selecting an appropriate optimizer, loss function, and performance metric.
4. Train the model using training data (x_train and y_train), specifying the number of epochs, batch size, and validation set split ratio.
5. Utilize the trained model for prediction to obtain predicted results (y_pred) for the test data.
6. Further analyze or apply the risk prediction results for research project risk management.
